# Complete Genome Sequence of the Nonmotile Myxococcus xanthus Strain NM

**DOI:** 10.1128/MRA.00989-21

**Published:** 2021-10-28

**Authors:** Dustin Joshua Vollmann, Tobias Busche, Christian Rückert, Markus Nett

**Affiliations:** a Department of Biochemical and Chemical Engineering, TU Dortmund University, Dortmund, Germany; b Technology Platform Genomics, Center for Biotechnology (CeBiTec), Bielefeld University, Bielefeld, Germany; University of Delaware

## Abstract

Myxobacteria exhibit multicellular swarming behavior, which depends on the coordination of cell motility. Unlike other myxobacteria, Myxococcus xanthus NM is not capable of forming swarms due to a defective motility system. Here, we present the 9.35-Mbp genome sequence of this nonmotile myxobacterium.

## ANNOUNCEMENT

Myxococcus xanthus is a bacterium that uses two types of locomotion (1, 2). Social (S) motility involves cooperative swarming movement, while adventurous (A) motility describes the gliding of single cells. M. xanthus strain NM originates from the type strain, FB, and is characterized by the loss of both motility systems (3, 4). In comparison to strain FB, NM was reported to be a superior host for the heterologous production of bioactive molecules (5). To clarify the genetic basis of its nonmotility, M. xanthus NM was sequenced.

M. xanthus NM was obtained from the American Type Culture Collection (ATCC 27925). After cultivation in liquid casitone-yeast extract (CYE) medium (6), genomic DNA was isolated using the NucleoSpin microbial DNA kit (Macherey-Nagel) with an additional RNase A (20 mg/ml) digestion (5 min, 70°C). Long and short DNA reads were generated by Nanopore and Illumina sequencing, respectively. For library preparation, a TruSeq DNA PCR-free high-throughput library prep kit (Illumina) and the SQK-LSK109 ligation sequencing kit (Oxford Nanopore Technologies [ONT]) were used without prior shearing of the DNA. To generate the short reads, a 2 × 300-nucleotide run (MiSeq reagent kit v3 [600 cycle]) was executed. The long reads were generated on a GridION platform using a R9.4.1 flow cell. Base calling and demultiplexing were performed using Guppy v5.0.11 in super high accuracy mode. The Illumina sequence data were assembled using Newbler v2.8 (7) (options: -large, -siom 16, -m, -consed) and quality filtered by screening against PhiX and adapter sequences. The Nanopore data were assembled using Canu v2.1.1 (8) (parameters: genomeSize = 15m, -rawErrorRate = 0.3, -correctedErrorRate = 0.1, ‘corMhapOptions=—threshold 0.8 —ordered-sketch-size 1000 —ordered-kmer-size 14’, -fast, readSamplingBias = 20). Reads shorter than 1 kb were excluded. Successively, Racon v1.4.16 (9) (parameters: -c 6, −m 8, −x −6, −g −8, −w 500), Medaka v1.4.3 (10) (parameters: -b 100, -m r941_min_sup_g507), and Pilon v1.22 (11) were used to polish the Canu contigs. This resulted in a single circular contig, which was confirmed using BLAST and Flye v2.9 (12). The *dnaA* gene was identified using BLAST, and the contig was split at this position prior to the final manual combination of the resulting assemblies in Consed v27.0 (13). Mapping was conducted using minimap2 v2.17 (parameters: -ax sr, –secondary_no), BWA-MEM v2 (14) (parameters: -O1, -E1), and Bowtie 2 v2.3.2 (15) (parameters: -X 750, –no-unal). The complete genome sequence was annotated using Prokka v1.14.5 (16). In total, 2 × 2,296,778 paired-end reads (Illumina) and 3,615,006 reads with an *N*_50_ length of 21.5 kb (ONT) were obtained. Based on the Canu parameters used, 97,106 reads were used for error correction.

The genome assembly comprises 9,346,321 bp, with a G+C-content of 68.88%. A nonsense mutation was found in the *agmX* gene by manual inspection of the miscellaneous features. Since AgmX is part of a multiprotein complex that is necessary for A motility in M. xanthus (17, 18), this mutation could partly explain the motility deficiency of strain NM.

### Data availability.

The whole-genome sequence was deposited at GenBank under the accession number CP080534. The raw data were submitted to the SRA under the accession numbers SRX11931648 and SRX11931649.
